# Minimally invasive Oxford medial unicompartmental knee replacement in patients 50 years of age or younger

**DOI:** 10.1007/s00402-022-04539-6

**Published:** 2022-07-18

**Authors:** Tilman Walker, Julius Stupp, Tobias Reiner, Benjamin Panzram, Timo A. Nees, Moritz M. Innmann, Tobias Gotterbarm, Christian Merle

**Affiliations:** 1 Department of Orthopaedic Surgery, University of Heidelberg, Schlierbacher Landstrasse 200a, 69118 Heidelberg, Germany; 2 Center for Orthopaedics and Trauma Surgery, University of Linz, Krankenhausstr. 7a, 4020 Linz, Austria

**Keywords:** Medial unicompartmental knee arthroplasty, UKA, UKR, Partial knee replacement, Oxford prosthesis, Mobile bearing, Young patients, Knee osteoarthritis

## Abstract

**Introduction:**

The aim of the present study was to assess clinical outcome and mid-term survivorship of mobile-bearing unicompartmental knee arthroplasty in patients 50 years of age or younger.

**Methods:**

This study reports the results of 119 patients (130 knees) following mobile-bearing medial UKA. Primary indication was advanced osteoarthritis or avascular necrosis of the femoral condyle. The anterior cruciate ligament (ACL) as well as the collateral ligaments were functionally intact, the varus deformity was manually correctable and there was no evidence of osteoarthritis in the lateral compartment. Survivorship analysis was performed with different endpoints and clinical outcome was measured using the Oxford Knee Score (OKS), American Knee Society Score and Functional Score (AKSS-O, AKSS-F), range of motion (ROM), Tegner activity score, University of California Los Angeles score (UCLA) and visual analogue scale for pain (VAS).

**Results:**

The survival rate was 96.6% at 6.5 years (95% CI 98.7–91.3%; number at risk: 56) and 91.7% (95% CI 96.7–80%; number at risk: 22) at 10 years for the endpoint device related revisions and 91.5% at 6.5 years (95% CI 95.4–84.5%; number at risk: 56) and 86.8% (95% CI 93–76.2%; number at risk: 22) at 10 years for the endpoint revision for any reason. Outcome scores, VAS and ROM showed significant improvements (*p* < 0.001). The mean OKS increased from 26.7 (standard deviation (sd): 7.2) preoperatively to 40.9 (sd: 7.6) at final follow-up, the mean AKSS-O from 48.3 (sd: 13.3) to 87.8 (sd: 14.4) and the mean ROM from 118° (sd: 16.7) to 125° (sd: 11.4). The radiological analysis revealed progression of degenerative changes in the lateral compartment in 39.6% of patients without affecting the functional outcome.

**Conclusions:**

Medial mobile-bearing UKA is a viable surgical treatment option in young patients with significant improvements in knee function and pain. Further follow-up is necessary to evaluate the long-term efficacy.

**Level of evidence:**

Retrospective cohort study, Level III.

## Introduction

Unicompartmental knee arthroplasty (UKA) is a highly effective treatment to restore knee function and reduce pain level in severe isolated medial arthritis of the knee joint. Compared to total knee arthroplasty (TKA) it is bone preserving and offers essential advantages, such as faster recovery, a better range of motion (ROM) with a more physiological knee kinematics, higher rates of satisfied patients and a lower peri- and postoperative morbidity and mortality rates [6, 18, 22, 29, 31].

For the Oxford mobile bearing prosthesis (Zimmer Biomet Inc., Warsaw, Indiana, USA) used in the medial compartment, excellent long-term results have been reported with survival rates of 98% after 10 and 91% after 20 years [20, 23, 30, 33]. Based on these encouraging results the indication for Oxford UKA (OUKA) has been extended to younger and more active patients [4, 7, 36]. However, knee arthroplasty in these patients remains challenging due to high expectations regarding postoperative knee function, level of activity as well as long life expectancy [15]. Thus, there is a major concern of a higher risk for revision surgery [10, 15, 16, 27] and an ongoing debate about the optimal treatment of very young patients with end-stage osteoarthritis in the knee joint [10, 26, 32]. Non-operative treatments, such as physiotherapy, bracing or anti-inflammatory medication are often limited in providing pain relief or functional improvement [26, 32]. Whereas, high tibial osteotomy (HTO) can be seen as an alternative option in patients with low-grade osteoarthritis and varus deformity, but it has been also demonstrated that the risk of failure increases for patients whose osteoarthritis is graded Ahlback grade 2 or higher [26]. In these patients, knee arthroplasty should be preferably considered [26].

Despite the growing interest of surgeons in UKA and the broadening of its indication to younger patients, there is only very few data about the clinical results and survival rates of UKA in this specific group of patients [3, 15, 16, 32], particularly in the group of patients younger than 50 years of age at time of surgery [10, 26].

Therefore, the purpose of this retrospective study was to evaluate clinical and radiological results as well as mid-term survivorship of minimally invasive medial unicompartmental knee arthroplasty in a large and independent series of patients 50 years of age or younger using a mobile-bearing prosthesis with a minimum follow-up of 12 months.

We hypothesized that medial OUKA ensures high functional outcome and good mid-term survivorship in this specific group of patients comparable to previously reported data for unselected patients regarding age at time of surgery.

## Patients and methods

This is a single-centre retrospective cohort study reporting the results of patients 50 years of age or younger following medial unicompartmental knee arthroplasty. Primary indication was advanced osteoarthritis of the medial compartment with full thickness articular cartilage loss (“bone on bone”) or avascular necrosis of the femoral condyle. In all cases, the anterior cruciate ligament (ACL) as well as the medial and lateral collateral ligaments were functionally intact, the varus deformity was manually correctable and there was no evidence of osteoarthritis in the lateral compartment on valgus stress radiographs. Osteoarthritis of the patellofemoral joint was not considered to be a contraindication unless there was deep eburnation or bone grooving on the medial facet of the patella. Rheumatoid arthritis, fixed varus deformity or a flexion deformity > 15° were considered to be a contraindication for UKA [35].

All UKA performed at the University Hospital of Heidelberg between September 2001 and March 2015 were screened for a possible inclusion into the study group. Inclusion criteria were 50 years of age or younger at the time of surgery as well as a minimum follow-up of 12 months. In all, 119 patients (130 knees) fulfilled the criteria and all of them were included into the study group. Patient demographics can be found in Table 1.

**Table 1:** Patients demographics

Total numbers of patients/knees	119/130
Follow-up (years) (mean, standard deviation)	6.5; 3.2
Age at time of surgery (years) (mean, standard deviation)	47.8; 2.6
Body mass index (kg/m^2^) (mean, standard deviation)	31.3; 5.9
Gender (%)	Male 59 (49%) 65 knees; Female 60 (51%) 65 knees
Operated side (%)	Right 64 (49%); Left 66 (51%);
Cemented/uncemented fixation (%)	105 (81%); 25 (19%)
Unilateral/bilateral (%)	108 (90.7%); 11 (9.3%)

The surgeries were performed by multiple surgeons using the Oxford partial knee arthroplasty (Zimmer Biomet Inc., Warsaw, Indiana, USA) with a minimally invasive surgical (MIS) technique and the prosthesis was implanted with cemented (81%) or uncemented fixation (19%). Before the introduction of the cementless version in 2009, all UKA were performed using a cemented fixation. Since then, the decision which kind of fixation should be used was based on patients bone quality. In both ways, immediate full weight-bearing was allowed post-operatively.

Survivorship analysis was performed for the endpoints “revision surgery” and “dislocation of the bearing”. The endpoint “revision for any reason” was defined as an operation in which at least one of the components was changed. Therefore, non-implant associated reoperations, such as debridement for hematoma or superficial wound healing delay without exchange of the mobile bearing were not considered as revision surgery. Furthermore, we differentiated the survivorship analysis according to the reason of revision surgery. The endpoint “device related revisions” was defined as specific complications of the implant or UKA, such as aseptic loosening, progression of arthritis and dislocation or breakage of the bearing, and the endpoint “aseptic revision” was defined as all revisions except for infection.

Clinical data were obtained as part of a regular check-up which were routinely performed for all patients 1, 3 and 5 years after joint replacement at our institution. In these, patients filled out questionnaires to receive patient reported outcome measures (PROMS), namely, the functional and objective American Knee Society Score (AKSS-F and AKSS-O) [12], the Oxford Knee Score (OKS) [5] as well as the Tegner- [2] and University of California Los Angeles score (UCLA) [38]. Pain level was noted by the patient using a visual analogue scale (VAS) ranging from 0 to 10 (0 = no pain to 10 = worst pain ever) and satisfaction with the prosthesis was rated with use of a numeric scale ranging from 1 (very satisfied) to 5 (unsatisfied). In addition, a clinical examination was performed by two of the authors (JS, TW) to obtain further information, such as range of motion (ROM), stability and knee-alignment. Patients who were not able to attend the clinical follow-up were contacted by telephone for a structured interview to fill out the questionnaires to assess the OKS, Tegner and UCLA score as well as satisfaction with the prosthesis, level of pain and possible complications. In patients with revision surgery, additional information was gathered from general practitioners, orthopaedic specialists or external hospitals for a better understanding of the circumstances leading to revision surgery.

Standardized postoperative radiographs were aligned with fluoroscopic control to obtain views parallel to the tibial component in the ap-view and parallel to the femoral component in the lateral view. The radiographs were independently analyzed by two examiners (JS, TW) focusing on radiological signs of loosening of the components and progression of osteoarthritis in the lateral compartment. Radiological signs of osteoarthritis in the lateral compartment were graded according to the Kellgren and Lawrence Score (KLS) [14] in the preoperative X-rays as well as in the most recent X-rays.

### Statistical analysis

Data were recorded and analyzed using SPSS version 17.0 (SPSS Inc., Chicago, IL) and Graphpad Prism version 5.0 (Graphpad Software, San Diego, CA). The empirical distribution of continuous variables was described using mean and standard deviation, possible differences between pre- and postoperative data were examined with the Wilcoxon Signed Rank test. Survivorship analysis was performed with the Kaplan–Meier estimator. For all tests, *p* values of < 0.05 were considered to be significant.

The institutional review board of the University of Heidelberg approved all procedures (S-068/2017) and the study was conducted in accordance with the Helsinki Declaration of 1975, as revised in 2013. Informed consent was obtained from all participating patients.

## Results

A total of 130 medial UKA were performed in 119 patients. The underlying diagnosis was isolated osteoarthritis of the medial compartment in 122 knees (93.7%) and avascular osteonecrosis of the femoral condyle in 5 knees (4%). In 3 patients (2.3%) osteoarthritis was posttraumatic due to a fracture of the tibia. In total, 81 knees (62.3%) had a history of previous arthroscopy of the knee joint for meniscectomy, joint lavage or cartilage surgery; in 10 knees (7.7%) corrective osteotomy was performed prior to knee arthroplasty. Two patients (2 knees) were lost to follow-up and three patients (3 knees) had died without need of revision surgery. A total of 12 patients (12 knees) had revision surgery for various reasons. The remaining 102 patients (113 knees) were reviewed at a mean follow-up of 6.5 years (sd: 3.2). From these, 82 patients (93 knees) were available for a clinical and radiological follow-up and 20 patients (20 knees) were available for a structured interview by telephone. 96.4% of the patients were reviewed with a follow-up of at least 2 years, 61.1% with a follow-up of at least 5 years and 18.6% with a follow-up of more than 10 years.

### Survivorship analysis

Revision surgery, defined as exchange or removal of at least one of the components was performed in 12 knees (9.2%). The most common indications for revision surgery were progression of osteoarthritis and persistence of pain in 3 patients (25%) each, followed by dislocation of the bearing in 2 patients (16.7%), infection or wound dehiscence in 2 patients (16.7%), aseptic loosening in 1 patient (8.3%) and mechanical complication in 1 patient (8.3%).

No revision surgery was performed due to progression of osteoarthritis in the patellofemoral joint. The reason for each revision surgery is described in Table 2.

**Table 2:** Summary of revision surgery

Patient number	Time to revision (years)	Reason for revision	Procedure
18	9	Aseptic loosening	Revision to TKA
30	7	Progression of lateral arthritis	Revision to TKA
33	0.1	Wound dehiscence/allergic reaction	Revision to TKA
46	3	Progression of lateral arthritis	Revision to TKA
48	2.9	Persistency of pain	Revision to TKA
49	0.8	Infection	Revision to TKA
65	1.2	Persistency of pain	Revision to TKA
68	5	Persistency of pain	Revision to TKA
98	0.3	Dislocation of the bearing	Bearing exchange
105	0.9	Dislocation of the bearing	Bearing exchange
111	0.03	Mechanical complication	Exchange of tibial component
119	1.6	Progression of lateral arthritis	Revision to TKA

Table demonstrating time to revision surgery, reason and the procedure performed for each revision

**Fig. 1:**
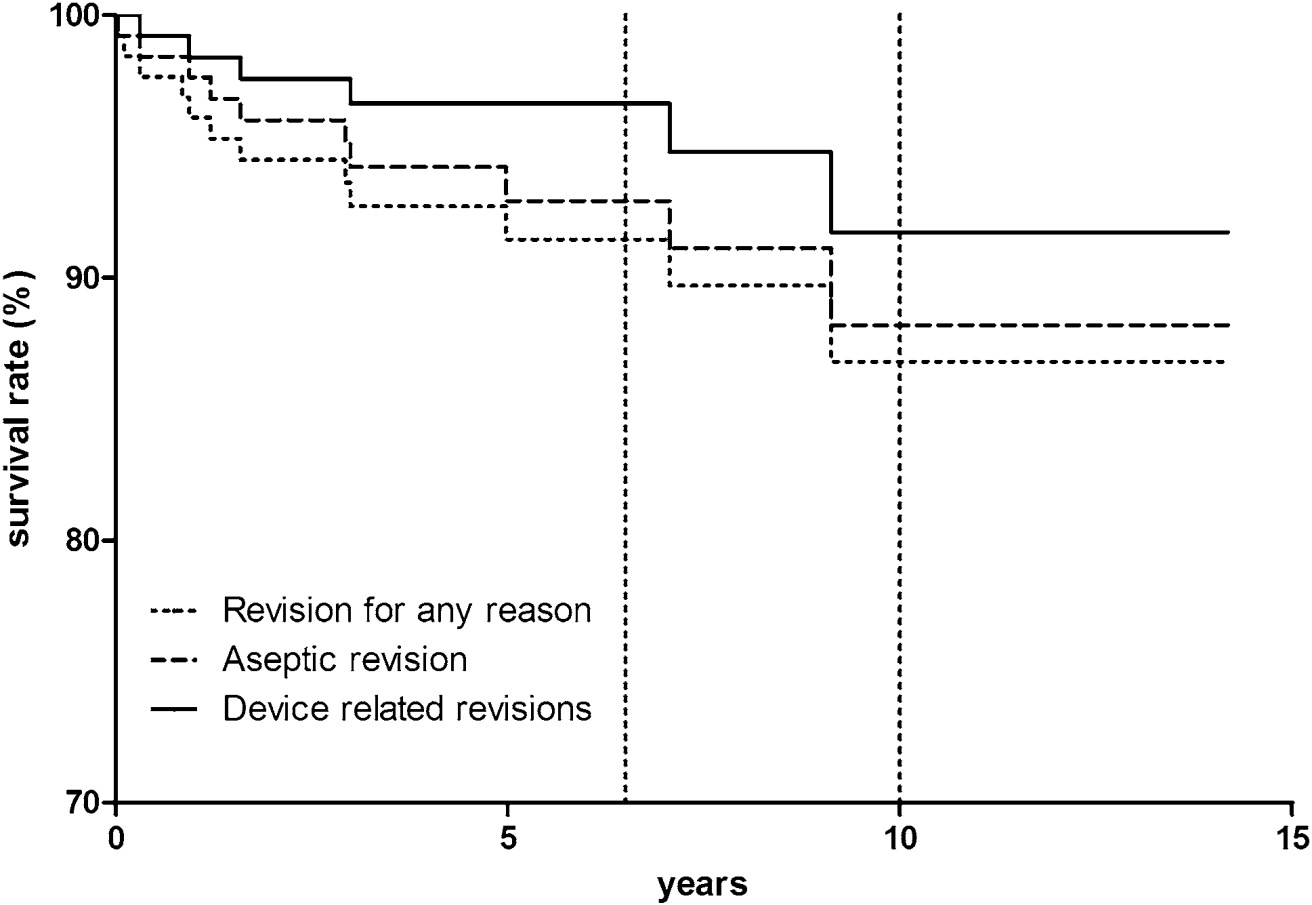
Kaplan–Meier survivorship for different endpoints: the 6.5-year survival was estimated at 96.6% (95% CI 98.7 to 91.3%, number at risk: 56), the 10-year survival at 91.7% (95% CI 96.7 to 80%, number at risk: 22) with the endpoint device related revisions. For the endpoint aseptic revision, the 6.5-year survival was estimated at 92.9% (95% CI 96.5 to 86.2%, number at risk: 56), the 10-year survival at 88.2% (95% CI 94.1 to 77.2%, number at risk: 22) and with the endpoint revision for any reason the 6.5-year survival was estimated at 91.5% (95% CI 95.4 to 84.5, number at risk: 56), the 10-year survival at 86.8% (95% CI 93% to 76.2%, number at risk: 22)

Kaplan–Meier survival analysis using device related revisions (aseptic loosening, progression of arthritis and dislocation or breakage of the bearing) as endpoint showed survival rates of 96.6% at 6.5 years (95% confidence interval (CI) 98.7 to 91.3%, number at risk 56) and 91.7% (95% CI 96.7 to 80%; number at risk: 22) at 10 years (Fig. 1). Regarding the endpoint aseptic revision, the survival at 6.5 years was 92.9% (95% CI 96.5 to 86.2%, number at risk: 56) and 88.2% (95% CI 94.1 to 77.2%, number at risk: 22) at 10 years and with the endpoint revision for any reason the survival at 6.5 years was 91.5% (95% CI 95.4 to 84.5; number at risk: 56 and 86.8% (95% CI 93% to 76.2%; number at risk: 22) at 10 years (Fig. 1). The cumulative incidence of bearing dislocation was 1.6% at both 6.5 (number at risk: 56) and 10 (number at risk: 22) years.

### Clinical outcome

**Fig. 2:**
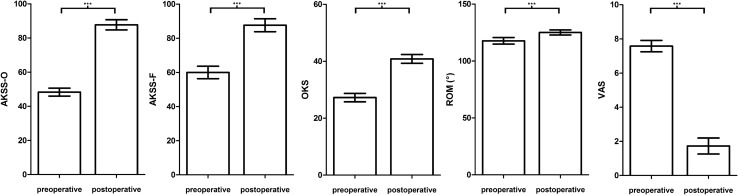
Clinical outcome: clinical outcome scores, range of movement and visual analogue scale for pain preoperatively and at minimum 12-month follow-up. The error bars represent the 95% Confidence Interval (CI), *** indicates *p* < 0.001; (OKS, Oxford Knee Score; AKSS-O, Objective American Knee Society Score; AKSS-F, Functional American Knee Society Score, ROM, range of movement; VAS, Visual analogue scale for pain)

The clinical results are demonstrated in Fig. 2 and Table 3. In all categories, the postoperative scores at final follow-up significantly improved when compared to preoperative values (*p* < 0.05) (Table 3). Prior to surgery, the mean OKS was 26.7 (sd: 7.2) and significantly improved to a mean score of 40.9 (sd: 7.6) postoperatively. According to the OKS-criteria, 58.2% of the patients had an excellent outcome (score > 41), 25.3% had a good outcome (34 to 41), 12.1% had a fair outcome (27 to 33) and 4.4% had a poor outcome (< 27) at final follow-up.

**Table 3:** Clinical outcome scores, range of movement (ROM), visual analogue scale (VAS) for pain preoperatively and at final follow-up

	Preoperative (mean, standard deviation,)	Postoperative (mean, standard deviation,)
Oxford Knee Score ***	26.7; 7.2	40; 7.6
Objective American Knee Society Score ***	48.3; 13.3	87.8; 14.4
Functional American Knee Society Score ***	60; 20.9	87.7; 18.2
Tegner Activity Score	n.a	3.6; 1.4
UCLA Activity Score	n.a	6; 1.7
Visual analogue scale pain ***	7.6; 1.9	1.7; 2.4
Range of movement (°) ***	118; 16.7	125; 11.4

*** indicates *p* < 0.001; Tegner Activity Score and UCLA Activity Score were not available (n.a.) preoperatively

The AKSS-O showed a statistically significant improvement from a mean score of 48.3 (sd: 13.3) preoperatively to a mean score of 87.8 (sd: 14.4) postoperatively. According to the AKSS-O criteria 72% of the patients had an excellent outcome (85 to 100), 15% had a good outcome (70 to 84), 4.3% had a fair outcome (60 to 69) and 8.6% had a poor outcome (< 60).

Preoperatively, the mean ROM was 118° (sd: 16.7) and improved statistically significant to a mean ROM of 125° (sd: 11.4). At final follow-up, 79.6% of the patients were able to flex the knee more than 120°. Physical activity was on a high level as demonstrated by the Tegner activity score and UCLA score (Table 3). Altogether, patients were satisfied or highly satisfied with the outcome of their implant in 94.3%, fairly satisfied in 4.7% and unsatisfied in 1%. When comparing pre- and post-operative pain levels, a significant improvement was shown (*p* < 0.05) (Table 3).

### Radiological outcome

No signs of loosening of the components were seen in the radiological analysis of those patients without revision surgery. According to the KLS, radiological signs of osteoarthritis in the lateral compartment grade I could be detected in 23% of our patients and grade II in 5% of our patients, preoperatively. There was no patient with an osteoarthritis grade higher than grade II. Postoperatively, 49% had signs of osteoarthritis grade I, 6% grade II, 4% grade III and 1.6% grade IV. In 39.6% of our patients, there were radiological signs of a progression of degenerative changes in the lateral compartment. Nevertheless, there was no statistically significant difference in the clinical outcome between patients with radiological signs of a progression of lateral arthritis and those without (*p* > 0.05).

## Discussion

The present study evaluated the mid-term follow-up results of patients 50 years of age or younger following medial mobile-bearing unicompartmental knee arthroplasty for end-stage osteoarthritis or avascular necrosis of the femoral condyle. The results confirmed our hypothesis that OUKA offers a high functional outcome and good mid-term survivorship in this specific group of patients. We could demonstrate high postoperative functional scores as well as a survival rate of 96.6% at 6.5 years and 91.7% at 10 years for the endpoint device related revisions and 91.5% at 6.5 years and 86.8% at 10 years for the endpoint revision for any reason.

To achieve good and reproducible results after UKA, it is necessary to define appropriate indications and contraindications [25]. In 1989 Kozinn and Scott were the first to develop specific disease- and patients-specific criteria for UKA [10, 17]. These criteria were based on outcomes of a series of 100 patients using a fixed-bearing device [10]. According to these authors, UKA should not be performed in patients who were younger than 60 years of age, weighed more than 82 kg, who were extremely physically active or performed heavy labour, had chondrocalcinosis or had exposed bone in the patellofemoral joint [17, 25]. In contrast to this, Goodfellow et al. recommended that these contraindications do not apply for mobile-bearing UKA [25]. With the aim to determine if the contraindications proposed by Kozinn and Scott should be applied to mobile-bearing UKA or can be ignored, Pandit et al. compared the outcome of patients with and without these criteria in a large series of 1000 Oxford UKA in 818 patients [25]. The cumulative 10-year survival rate in the group of patients without any of the proposed contraindications was 93.6% compared to 97% in the group of patients with at least one of the contraindications [25]. Regarding the criterion of age, the survival rate at 10 years was 97.3% in the group of patients less than 60 years compared to 95.1% in the group of patients more than 60 years of age [25]. These results could be confirmed in a more recent study by Hamilton et al. [11, 25]. Based on these results, it is believed that age and the further mentioned patient-specific contraindications do not apply to the used mobile-bearing UKA, potentially due to its specific implant characteristics [11].

Nevertheless, joint replacement in young patients remains a particular challenge for surgeons [1, 8]. A high level of activity in combination with high and partly unrealistic expectations concerning the ability to return to sporting activities after knee or hip arthroplasty, may lead to dissatisfaction even after a technically successful procedure [8, 36, 37]. Furthermore, the limited lifespan of an implant and the higher risk of revision surgery are major concerns in these patients as the risk of complications increase with each reoperation and the decreased survivorship seen in revision surgery especially affects younger patients [1, 8, 26, 36].

In general, the survival rates of UKA in young patients are heterogeneous in current literature.

In a data analysis of the Australian and Swedish knee registries, Dahl et al. were able to demonstrate a statistically significant increase in the cumulative revision rate (CRR) with decreasing age [34]. At 7 years, the CRR of patients younger than 55 was 19% compared to 5.7% in patients older than 75 years [34]. In a multicenter study by Price et al. the survival rates of 403 patients (512 knees) older than 60 years were compared to 44 patients (52 knees) younger than 60 years following OUKA [28]. In their study, the 10-year survival for patients older than 60 years was 96% compared to 91% for patients younger than 60 years [28]. Similar survival rates of 92.8% after 10 years could be demonstrated by Kim et al. in a series of 80 patients with a mean age of 54 years following 106 OUKA [15] and by Faour Martin et al. with a survival rate of 95% at 12 years in a series of 55 patients under the age of 60 years. In another series of 46 patients following OUKA, Kort et al. reported two revisions at a maximum follow-up of 6 years [16]. In a study of 62 patients with a mean age of 55 years, Felts et al. demonstrated a survival rate of 94% after 12 years using a fixed bearing device [8] and Pennington et al. a survival of 92% after 12 years in 46 knees with a mean age of 56 years using a fixed bearing device as well [27].

Although there are a number of studies focussing on the survival of UKA in patients less than 60 years of age as described above, data on the outcome of patients less than 50 years of age following UKA is rare.

Parratte et al. reported the results of a series of 35 UKA in 31 patients with a mean age of 46 at the time of surgery using a cemented fixed-bearing prosthesis (Miller-Galante, Zimmer, Warsaw, Indiana) [26]. Patients demonstrated significant improvements in the functional outcome scores and 91% of the patients were satisfied or highly satisfied with the knee function. In total, six knees required revision surgery resulting in a survival rate of 80.6% at 12 years with revision for any reason as endpoint. The reason for revision surgery was wear of the polyethylene inlay in four patients, one knee was revised for aseptic loosening and one for progression of arthritis [26]. There is only one previous study demonstrating the clinical results and survival rates of patients following mobile-bearing UKA under the age of 50 years. Greco et al. reported the results of 340 knees in 279 patients with a mean age of 46.5 years using the Oxford partial knee arthroplasty [10]. At a mean follow-up of 6.1 years, clinical function scores improved significantly as well as patients’ activity measured by the UCLA-Score. A total of 20 revision surgeries were performed, resulting in a survival rate of 96% at 6 years and 86% at 10 years using all-cause revision surgery as endpoint [10], which is similar to the results in our study.

The clinical results and patient reported outcome measures in the present study are good to excellent and comparable to those previously reported in the literature for unselected patient groups. Pandit et al. demonstrate an AKSS-O of 92, an AKSS-F of 80 and a mean flexion of the knee joint of 133° in their series of 1000 OUKA, Lisowski et al. could demonstrate an OKS of 42 and an AKSS-O of 81 [19, 24]. With the results of the Tegner activity score as well as the UCLA-Score, it could be demonstrated that most patients are physically active in their daily routine and participate to some degree in sports or physical activities. Similar results were demonstrated by several previous studies on mobile bearing UKA [9, 21, 36]. Even if in 39.6% of our patients a progression of degenerative changes in the lateral compartment could be detected according to the KLS, there was no impact on the clinical outcome in these patients. Most of these patients only had mild changes with a KLS grade I or II which might be seen as physiological due to an increased age. Similarly, Jiao et al. were able to demonstrate that even the presence of slight cartilage damages (Outerbridge grade 1 or 2) in the weight-bearing area of the lateral femoral condyle did not compromise the short-term outcome of medial mobile-bearing UKA according to the OKS and patients satisfaction [13]. Nevertheless, progression of arthritis remains the most common reason for revision surgery and further studies should focus on factors which might influence this progression.

The major limitations of the present study include the retrospective study design, the relatively short clinical follow-up period with a minimum follow-up of 12 months and a mean follow-up of 6.5 years as well as the relatively small number of patients. In addition, 20 patients were only available for a structured interview by telephone and this study does not have a control group of patients 60 years of age or older allowing for a direct comparison of the clinical results and implant survival. The main strengths of this study are that only < 2% of the patients were lost to follow-up and all patients received the same postoperative rehabilitation regime. In addition, this study was completed in an independent centre and there is only one study demonstrating similar results.

## Conclusions

The present study demonstrates significant improvement in knee function and pain relief in patients 50 years of age or younger following mobile-bearing UKA in the medial compartment. The survival rate as well as the clinical outcome are comparable to those previously published for unselected patient groups. Therefore, OUKA can be seen as a viable surgical treatment option in young patients with end-stage osteoarthritis of the medial compartment. Nevertheless, further follow-up is necessary to evaluate the long-term effectiveness of this device in this group of patients.
